# Impact of Vitamin D Supplementation on Multiple Sclerosis

**DOI:** 10.7759/cureus.18487

**Published:** 2021-10-05

**Authors:** Fenil Gandhi, Sharan Jhaveri, Chaithanya Avanthika, Abhishek Singh, Nidhi Jain, Azouba Gulraiz, Pratiksha Shah, Fareeha Nasir

**Affiliations:** 1 Internal Medicine, Shree Krishna Hospital, Anand, IND; 2 Internal Medicine, Nathiba Hargovandas Lakhmichand Municipal Medical College, Ahmedabad, IND; 3 Medicine and Surgery, Karnataka Institute of Medical Sciences, Hubli, IND; 4 Pediatrics, Karnataka Institute of Medical Sciences, Hubli, IND; 5 Internal Medicine, Mount Sinai Morningside, New York City, USA; 6 Internal Medicine, Sir Ganga Ram Hospital, New Delhi, IND; 7 Medicine, California Institute of Behavioral Neurosciences and Psychology, Fairfield, USA; 8 Vascular Surgery, Shree Krishna Hospital, Anand, IND; 9 Internal Medicine, Harlem Hospital Center, New York City, USA

**Keywords:** internal medicine, chronic illness, multiple sclerosis, public awareness of vitamin d, relationship between diseases and nutrition, vitamin d supplementation, neurology and systemic disease, relapsing-remitting multiple sclerosis, general internal medicine

## Abstract

Multiple sclerosis (MS) is an autoimmune disease affecting a large number of people every year. The exact causal factor for this disease is unclear, but it commonly affects middle-aged women, with known triggers like stress, childbirth, infections, poor diet, lack of sleep, etc.

Many epidemiological studies have indicated that various genetic abnormalities are also critical drivers of the onset of MS. The major risk factors of MS identified include hypovitaminosis D while environmental protective factors include allele HLA DRB1 1501, obesity, Epstein-Barr virus infection, sexual hormones, and smoking.

Our article explores the correlation between the deficiency of vitamin D and the onset and progression of MS. The study uses a systematic review methodology by researching and reviewing scholarly articles exploring the topic. We conducted online searches of literature on Google Scholar and PubMed using the keywords "vitamin D deficiency" and "multiple sclerosis" and accessed the relevant secondary literature sources for review. The variables under study included vitamin D insufficiency as the dependent variable while MS was the independent variable. Causal variables included environmental, genetic, and protective factors.

We hypothesized that there is indeed a correlation between vitamin D deficiency and MS. The findings from our review indicate a strong correlation between the insufficiency of vitamin D and the onset and progression of MS. These results are essential in devising interventions to accomplish primary and secondary prevention of MS, as well as integrating vitamin D supplementation in current treatment protocols for MS.

## Introduction and background

Vitamin D is a fat-soluble hormone that can be characterized in ergocalciferol and/or cholecalciferol. It is acquired mainly from dietary intake and by exposing skin to sunlight. D3 (cholecalciferol) is acquired from exposure of the skin to the sun and ingestion of fish, milk, and plants; D2 (ergocalciferol) is acquired from the ingestion of plants. Both are converted to 25-hydroxyvitamin D and are stored in the liver, which then gets activated into 1,25-hydroxyvitamin D in the kidney. Vitamin D also plays a vital role in the absorption of calcium [1-3]. Many clinical studies have established a relationship between vitamin D levels and the exacerbation of multiple sclerosis (MS) [4].

MS is a chronic, progressive disease that causes autoimmune inflammation and demyelination of the central nervous system (CNS) with subsequent axonal damage. It can present as acute optic neuritis (most common), brainstem/cerebellar syndrome, pyramidal tract demyelination, and/or spinal cord syndromes. Attacks of MS are characterized by asymptomatic episodes that are separated in time and space. Commonly described as having a "relapsing and remitting" clinical course, these are symptomatic episodes that occur months or years apart and affect different parts of the CNS [1].

MS is depicted by inflammation with demyelination, extensive immune infiltration, damage to oligodendrocytes, and axonal loss, supposedly autoimmune in nature [5]. Most often, it affects women in their 20s and 30s and is most commonly seen in individuals living farther from the equator [6]. Deficiency of vitamin D is also common in temperate areas due to a lack of sunlight and altered lifestyles [7]. Both sun exposure and vitamin D level, independent of serum levels, are linked to multiple sclerosis [8].

Sufficient vitamin D levels have decreased the prevalence and progression of MS. The role of vitamin D in the pathogenesis of MS is not entirely understood; however, some genetic studies have shown that various immunomodulators associated with MS are linked to vitamin D-associated regulation of gene expression [8]. This article aims to assess the correlation between the clinical efficacy of vitamin D supplementation and symptom control in patients with MS.

## Review

Epidemiology

Multiple sclerosis is considered one of the leading causes of neurological disability in adults. Global prevalence rates range anywhere from 50 to 300 per 100,000, with approximately 2.3 million cases worldwide [9]. Prevalence was noted highest in Northern America and Europe and lowest in Sub-Saharan Africa and Eastern Asia. Due to the relative lack of data in the large populations of India and China, this is widely regarded as a gross underestimate [10]. However, there has been an increase in reporting in recent years, with studies from the Middle East, North Africa, and Southwest Asia reporting increasing prevalence over the last few years [11].

Despite receiving more attention in recent years, Posner and Brinar noted that the data on multiple sclerosis can be misleading as reliance on clinical information and brain MRI interpretation is responsible for one-third of incorrect MS diagnoses [12]. According to them, the country of training or specialization in MS did not influence the results, whereas misdiagnosing disseminated encephalomyelitis as MS may have led to falsely elevated prevalence rates.

A recent meta-analysis by Simpson S Jr. et al. evaluated data from 59 countries and found a strong North-South gradient for MS prevalence and incidence, with a statistically significant increase as one moves away from the equator [13-15]. However, this was in stark contrast to other studies that found no such gradient in the prevalence of MS [16-17]. This hypothesis of increasing prevalence and incidence was based on the theory of sunlight exposure, and irrespective of the debate surrounding it, this gradient is attenuating, especially in the US [18]. Higher vitamin D levels and hygiene improvements are some of the theories proposed for this recent change. This hypothesis is further supported by the lower prevalence of MS in the coastal villages of Norway, where vitamin D-rich fish consumption seems to have a lower prevalence of MS as compared to inland villages placed at similar latitudes [19].

In most populations, the female to male ratio for MS lies between 1.5:1 to 2.5:1, with recent studies reporting higher values. This may be due to several factors. First, women tend to be more susceptible to autoimmune conditions in general, an observation that is most likely related to hormones such as estrogen and progesterone because of their concentration-dependent effects on the immune system. Second, some studies have proposed a change in smoking habits to be a major cause of a higher female to male ratio [20]. Thirdly, women are more likely to seek medical help for benign symptoms, which are seen in a majority of MS cases and can be easily diagnosed with the new diagnostic criteria [18].

​​Pathophysiology

Multiple sclerosis is somewhat of an enigma in medicine. Genetic susceptibility seems to be a well-established risk factor for MS. With genome-wide association studies becoming popular, more than 100 genetic regions have been linked with MS [21]. However, this is not the case with the other category of risk factors, environmental influences. Complex confounding data has been a barrier in establishing environmental risk factors for MS [22].

Although many theories attempt to explain the progression of MS, the primary trigger remains unknown. The current literature agrees on the fact that MS is an autoimmune process directed towards myelin antigens, such as gangliosides, myelin basic protein, proteo-lipoproteins, etc. [5,22-23].

The main driver of this disease seems to be the adaptive immune system although some studies suggest the opposite, i.e., the innate immune system playing a primary role in the initiation and progression of the disease [24]. Irrespective of the agent involved, the development of the disease can be explained based on two models, which co-relate well with the relapsing-remitting clinical course seen in a majority of cases.

In general, these can be divided into CNS extrinsic and CNS intrinsic theories [25].

The CNS Intrinsic Theory

According to this theory, MS develops and is propagated due to CNS intrinsic events, and autoreactive lymphocytic infiltration is a secondary phenomenon. The primary phenomenon here is still unknown and has been postulated to be related to primary neurodegenerative processes like Alzheimer's or even a reaction to an undiscovered viral infection [26-27]. These events would cause the release of CNS antigens to the periphery (by antigen-presenting cells or by drainage due to damage to the blood-brain barrier secondary to inflammation), thus generating an auto-immune response.

The CNS Extrinsic Theory

Molecular mimicry, T cell co-expression, or bystander activation of T cells have all been proposed as possible inciting events. The activated B cells and monocytes reach the CNS by crossing the blood-brain barrier or blood CSF barrier via the choroid plexus and propagate inflammation there [27]. This model is also used to study MS-like diseases by introducing activated cells into the CNS of animals, thus kick-starting the disease.

The hallmarks of MS lesions include axonal loss, astrocytic gliosis, demyelination, and plaque formation. Axonal loss is thought to be due to inflammatory mediators, such as reactive oxygen species (ROS) and NO, released from activated inflammatory cells, ultimately leading to mitochondrial dysfunction [28]. This further propagates ROS generation and contributes to demyelination. Clinically, these disease processes have wide-ranging implications. For instance, both of the above mechanisms, ROS generation and demyelination, are responsible for the visual loss that occurs with optic neuritis, a typical finding in MS [29]. Moreover, demyelinated axons may become hyper-excitable, thus being responsible for the positive symptoms that occur; or on the other hand, demyelination may slow conduction and cause ectopic signals [30].

With respect to vitamin D, there are strong indicators of it having a critical role to play in immunomodulation. Higher levels of vitamin D, irrespective of dietary intake, seem to predict a lower risk of MS [31]. Moreover, children born with low vitamin D levels and insufficient vitamin D levels during pregnancy are both associated with higher risks of MS [32-33]. There have been studies that have found contradictory findings, however, whether they should be considered conclusive is debatable [34].

Vitamin D as an immunomodulator

Vitamin D is undoubtedly important in the maintenance of calcium and phosphate levels in the body, as well as in bone metabolism. Evidence suggests that immune cells unregulated convert 25(OH)D to 1,25(OH)2D and that they are dependent on 25(OH)D levels in the blood that are at least 30 ng/mL (75 nmol/L) [35-37]. If 1,25(OH)2D is produced, it acts in both an autocrine and a paracrine way to control both the adaptive and innate immune systems, respectively. In addition, there is some indication that vitamin D, via the maintenance of endothelium membranes, may control immune activation in a non-genomic manner [38]. In the current state of knowledge, the vast majority of evidence suggests that maintaining a healthy vitamin D level is critical for regulating the body's immune activity. Low serum 25(OH)D levels have been associated with a variety of immune-related ailments, including autoimmune disorders and infectious diseases, according to recent research. Despite the exceptions reported in this research, there is less compelling proof that vitamin D is a useful treatment strategy for autoimmune illnesses and infectious diseases than there is for other conditions. Whether vitamin D therapy is beneficial as an extra immunomodulatory medication in the treatment of the majority of diseases is still up for debate based on contradictory clinical trial findings.

Efforts to raise public awareness about the health benefits of vitamin D, along with policies to fortify frequently consumed foods with vitamin D, should be undertaken to minimize the chances of vitamin D deficiency throughout pregnancy, childhood, and young and middle-aged adulthood, when autoimmune disorders are the most prevalent. Furthermore, increasing vitamin D status from birth to death may contribute to a reduction in the prevalence of viral diseases, such as influenza and COVID-19, which may have serious consequences, especially for the older population. But additional studies are needed to determine who would benefit the most from vitamin D and how much vitamin D is required for optimal health benefits based on each individual's vitamin D responsive profile, among other things. It is also uncertain whether or not giving 1,25(OH)2D3 or one of its analogs is a feasible therapeutic option for autoimmune disorders and infectious diseases such as influenza. When blood levels of 1,25(OH)2D3 rise significantly, intestinal calcium absorption increases, which, if left uncontrolled, leads to hypercalciuria and, ultimately, hypercalcemia. This is why blood levels of 1,25(OH)2D3 must be constantly monitored. Because of this, it is more likely that the immunomodulatory effects of vitamin D are attributable to the production of 1,25(OH)2D by immune cells such as monocytes and macrophages.

However, even though the bulk of vitamin D's biological actions have been attributed to its active metabolite, there is strong evidence that vitamin D has its own biological activities that are distinct from those of its active metabolite. Our hunter-gatherer forefathers and foremothers most likely had vitamin D levels in their blood in the 10-50 ng/mL (25-125 nmol/L) range. This result is supported by the observation that Maasai herders and Hadza tribespeople maintained serum 25(OH)D levels in the 40-60 ng/mL (100-150 nmol/L) range [39-40], respectively. A person would need to take 4000-6000 IUs of vitamin D each day in order to maintain these blood levels. Thus, circulatory vitamin D concentrations would stay between 20 and 40 ng/mL (50 and 100 nmol/L) for the foreseeable future. Finding that in vitro vitamin D3 was significantly more effective than either 25(OH)D3 or 1,25(OH)2D3 at stabilizing endothelial membranes and thereby decreasing inflammatory response may help explain the intriguing clinical findings that extremely high doses of vitamin D have been effective in treating or at least reducing symptoms of some autoimmune disorders, such as psoriasis, vitiligo, and multiple sclerosis, among other conditions. A recent study found that giving 60,000 international units of vitamin D once a day for 10 days helped children with congenital autosomal recessive ichthyosis and epidermolytic ichthyosis drastically improve. This finding lends support to the notion that vitamin D may be essential in the preservation of good health [41]. There are still unresolved questions that need to be investigated further in order to fully utilize the immune system's beneficial effects of vitamin D in clinical practice. The conclusion is that increasing our vitamin D intake to maintain blood 25(OH)D levels of at least 30 ng/mL (75 nmol/L), and preferably 40-60 ng/mL (100-150 nmol/L) for optimal overall health benefits, is not associated with any negative consequences.

Vitamin D is well-known for its conventional hormonal action in regulating mineral and skeletal balance, which has been well-documented. The discovery that the vitamin D receptor (VDR) is expressed in the vast majority of non-skeletal tissues, on the other hand, demonstrates the broad range of functions it has in the human body. Vitamin D receptor (VDR) expression in non-skeletal tissues and dietary vitamin D has many different functions, and the present study emphasizes these functions, with a special focus on its immunomodulatory properties. Because VDR and the enzyme 1-hydroxylase are expressed in the vast majority of immune cells, vitamin D has an effect on the phagocytic activity of macrophages and natural killer cells (NK cells). Furthermore, it increases the activity of phagocytes that are involved in microbicidal defense. Antigen-presenting dendritic cells and B lymphocytes are inhibited in their differentiation and maturation by vitamin D while Th1 and Th17 cells are inhibited in their proliferation by vitamin D [42].

As with type 1 diabetes, multiple sclerosis (MS) is much more prevalent in countries with higher latitudes, where people are more susceptible to vitamin D deficiency [13]. Living south of 35° latitude during one's first 10 years of life is associated with a 50% lower risk of developing multiple sclerosis [43]. A prospective nested case-control study of 148 MS patients and 296 controls showed that every 20 ng/mL (50 nmol/L) increase in blood 25(OH)D levels over 24 ng/mL (60 nmol/L) decreased the incidence of MS by 41% (odds ratio, 0.59; 95% confidence interval, 0.36-0.97) [31]. In the same study, researchers discovered that women who took more than 400 IUs of vitamin D each day had a 41% reduced risk of developing multiple sclerosis [1]. Because of this, vitamin D deficiency is thought to play a role in the formation of dysregulated T helper cells, cytotoxic T lymphocytes (CTLs), natural killer cells (NK cells), and B cells in the central nervous system, ultimately leading to the autoinflammation of the central nervous system that injures neurons and oligodendrocytes noted in MS [38,44].

Individuals who have particular human leukocyte antigen (HLA) alleles, such as HLA-DRB1*1501, have a significantly higher chance of developing multiple sclerosis [45-46]. According to the findings, vitamin D response elements have been discovered in the promoter region of the HLA-DRB1 gene, and the gene's expression may be altered by activation of VDR by 1,25(OH)2D, thus strengthening the link between vitamin D and MS [47-48].

Many of the actions of 1,25(OH)2D on the immune system are similar to those described for interferon-beta, an immunomodulatory medication used to treat multiple sclerosis (MS). This suggests that vitamin D may have a therapeutic function in the treatment of MS. The results of current randomized controlled trials are conflicting; however, some studies have found that high-dose vitamin D supplementation (up to 14,000 IUs/day) alone or as an add-on treatment has a significant effect on decreasing the relapse rate and improving inflammation markers as well as abnormalities on magnetic resonance imaging (MRI) in people with MS. According to the results of one meta-analysis performed by McLaughlin et al. to examine the function of therapeutic vitamin D in multiple sclerosis (MS), the final analysis included 12 studies with a total of 950 participants. A PubMed database search was carried out to locate clinical trials assessing vitamin D in people suffering from relapsing-remitting multiple sclerosis (RRMS). Using inclusion and exclusion criteria, the studies were selected for inclusion. Because of the wide range of study designs, the papers were divided into four groups for further analysis. Except for three studies, all were found to have a low or unclear risk of bias, as shown by funnel plot analysis. In any of the outcome indicators, there was no statistically significant difference between the groups. There were non-significant trends in favor of vitamin D across all outcome markers, particularly when only placebo-controlled studies were included. A significant increase in the annualized relapse rate (mean difference 0.15 [95 percent confidence interval: 0.01-0.30]), as well as non-significant trends of increasing Expanded Disability Status Scale and gadolinium-enhancing lesions, were observed in the higher-dose arms, according to the results of dose-comparison studies. This suggests that vitamin D supplementation may have a therapeutic role in the treatment of multiple sclerosis based on the findings. Although there is considerable disagreement regarding the optimal dosage, high dosages may result in less effective outcomes. Vitamin D studies in multiple sclerosis (MS) that are well-conducted and randomized with dosage ranges and placebo controls are still needed [49]. The authors would like to point out that the majority of existing clinical studies included a small number of patients, and that the vitamin D dosages utilized for treatment varied significantly from study to study.

Brazil's clinical research program has been conducting studies with very high dosages of vitamin D3 in order to cure a variety of autoimmune illnesses, including psoriasis, vitiligo, and multiple sclerosis [50]. In five MS patients who had either failed to respond to or refused conventional MS treatment, treatment with a very high dose of vitamin D supplementation (50,000 IUs/day or 1000 IUs/kg/day) to increase serum 25(OH)D level to 200-300 ng/mL (500-750 nmol/L) was found to be remarkably effective in controlling and improving symptoms as well as improving MRI findings. It was shown that urging patients to adhere to a low calcium diet more closely decreased the chance of developing hypercalcemia and hypercalciuria. That meant that all dairy products, as well as any other meals containing significant amounts of calcium, were to be eliminated from the diet. One study found that giving a 52-year-old female MS patient 40,000 IUs/day (1000 IUs/kg/day) of vitamin D3 for five years significantly reduced her neurological symptoms. A calcium-containing meal was prohibited, and she was told to follow this rule. Her serum 25(OH)D levels were constant at about 250 ng/mL (625 nmol/L), although her total calcium levels temporarily increased around the time of her diagnosis. Following a review of her eating habits, it was discovered that she was consuming a significant amount of calcium-rich vegetables. That calcium was eliminated from her diet, and that her blood calcium levels returned to normal, where they have stayed for the last five years, shows that calcium was removed from her diet. During the first year of life, blood PTH levels were in the low normal range while serum 1,25(OH)2D levels were above the normal range. They both returned to normal after the diet modification and have stayed within the normal range ever since. Neither hypercalciuria nor kidney stones nor nephrocalcinosis could be detected in this study. Similarly, Michael F. Holick et al. saw a rise in blood calcium and calciotropic hormone levels in a 32-year-old man who refused conventional therapy and was given 54,000 IUs of vitamin D3 daily in another study. The 25(OH)D level in his bloodstream rose rapidly within two months, reaching about 250 ng/mL (625 nmol/L) after two months of intensive treatment. His serum PTH and 1,25(OH)2D levels had stayed normal during the four-month period, as had his 24-hour urinary calcium excretion [51].

A randomized controlled study (RCT) investigating the efficacy and safety of a high-dose vitamin D supplement (1000 IUs/kg/day) for multiple sclerosis (MS) would be a perfect start to this interesting hypothesis. The use of lower vitamin D doses (up to 14,000 IUs/day) as a supplement seems to offer some benefits in terms of disease activity, even though the evidence is weak [51]. What is understood is that keeping a healthy 25(OH)D level in the blood and consuming enough amounts of vitamin D may reduce the risk of developing MS in certain people. More study, however, is required before this treatment strategy can be implemented in routine clinical practice.

Diagnostic and treatment protocols currently in use for multiple sclerosis (MS)

To diagnose MS, doctors now utilize the McDonald criteria, which is based on the development of CNS lesions across time and space [52].

Dissemination in time (DIT) is defined as the formation of new lesions over a period of time, as shown by one or more of the following tests:

At least two exacerbations that occur no more than 30 days apart are required.

If an MRI is performed at any point in time, it may show the presence of both gadolinium-enhancing and non-enhancing lesions, as well as the development of a new hyperintense T2 or enhancing lesion on subsequent MRI scans [52].

CSF oligoclonal bands are present in the absence of serum oligoclonal bands, which is an alternate test to DIT (indicate ongoing intrathecal inflammation).

Dissemination in space (DIS) is the presence of lesions in several regions of the central nervous system (CNS), which may be confirmed by one of the following tests:

Having two or more lesions that are substantiated by objective clinical data is considered present.

In the clinical setting, the objective clinical evidence of a lesion is defined as the relationship between a patient's symptoms and objective results (for example, T2 hyperintensities in the regions corresponding to the somatosensory tracts on magnetic resonance imaging or abnormal somatosensory-evoked potentials in a patient who reports sensory loss).

One or more hyperintense lesions on magnetic resonance imaging (MRI) in T2 sequence in at least two of the following areas: spinal, periventricular, juxtacortical, and infratentorial are some of the categories adopted.

Instrument-based diagnostics [53-56]

Plain MRI (of the brain and spine) is the recommended method of examination. Multiple sclerotic plaques with finger-like radial extensions (Dawson's fingers) that are linked with demyelination and reactive gliosis are seen in patients (most frequently observed in periventricular white matter).

As well as black-hole lesions, hypo-/isointense demyelination and axonal degeneration are seen in T1. Numerous hypointense lesions are referred to together as "black-hole lesions." The presence of black-hole lesions is associated with a poor prognosis. T2 and FLAIR are hyperintense.

Contrast MRI (with gadolinium): active lesion enhancement throughout the course of the exacerbation and for up to 6 weeks following it.

Slower conduction of the optic nerve and a longer latency in the occurrence of visual evoked potentials. Despite the fact that MRI scans have largely supplanted electrophysiological examinations, VEPs may still detect latent lesions in approximately 70% of individuals when they are performed. Other types of evoked potentials (such as auditory and somatosensory) are not indicative of the condition.

The CSF is found to have a pleocytosis lymphocytic pattern on examination. Oligoclonal bands (IgG subfraction formation) are also present: the presence of many oligoclonal bands in CSF and the absence of these bands in blood are both highly suggestive of multiple sclerosis (MS). The development of oligoclonal bands on electrophoresis or isoelectric focusing of CSF in the context of MS is possible. As a result of intrathecal inflammation, these bands demonstrate an increase in the production of many nonspecific clones of IgG inside the central nervous system (CNS) (unlike monoclonal gammopathy in cases of multiple myeloma). In addition, myelin basic protein levels in the CSF have risen. Please keep in mind that when oligoclonal bands appear in the early stages of the disease, this indicates a poor prognosis.

Treatment of multiple sclerosis

The goal is to begin treatment as soon as possible in order to treat the first exacerbation, prevent future exacerbations, and slow the development of the illness.

For acute exacerbation, high-dose intravenous glucocorticoids are the first line of therapy (methylprednisolone). Plasmapheresis is the second line of treatment. Patients who do not react to or tolerate corticosteroid medication may benefit from the use of adrenocorticotropic hormone (ACTH) gel as an alternative treatment. Through the melanocortin system, the melanocortin peptide ACTH has direct anti-inflammatory and immune-modulatory actions, while also indirectly increasing cortisol production through the adrenal cortex. ACTH has deleterious effects that are similar to those of corticosteroids, but it may be less destructive to bone and may be associated with a lower incidence of avascular necrosis (AVN) [57].

While the management of an acute exacerbation of multiple sclerosis is rather simple, the therapy of multiple sclerosis over the long term is dependent on the kind of MS that the patient is experiencing. Primary progressive MS (PP-MS) is the most severe of the four types of MS that have been identified: Clinically isolated syndrome (CIS), relapsing-remitting MS (RR-MS), secondary progressive MS (SP-MS), and secondary remitting MS (RS-MS).

People having their first episode of MS who are at high risk of acquiring MS, as well as those that have been diagnosed with RR-MS, should be treated as a matter of urgency [52].

Rather than using high-dose interferon-beta-1a and glatiramer acetate as first-line therapies for the majority of individuals with active MS, it is now advised that they utilize highly effective disease-modifying medications [58]. In contrast to the traditional "treat to target" approach, in which therapy of modest or moderate effectiveness would be initially used and advanced to a more effective agent when breakthrough disease (as determined clinically or by MRI) occurs, the new recommended approach makes use of treatment of high effectiveness from the beginning of the treatment process. Observational studies have shown that initiating high-efficacy therapy early in the course of the disease improves long-term outcomes in the patient. In the vast majority of cases, we suggest beginning therapy with ocrelizumab or another anti-CD20 drug, or with natalizumab in individuals who do not have the John Cunningham virus, before moving on to other options. Therapy with anti-CD20 antibodies is a potential treatment option because of their high degree of efficacy, low frequency of infusions or injections, favorable safety profile, and absence of rebound following treatment discontinuation. Individuals who have new or growing MRI lesions as a result of progressive therapy multiple sclerosis (PPMS) may also be considered for ocrelizumab treatment. The following situations may necessitate a change in therapy: suboptimal response, having experienced more than one relapse with active MRI scans in the prior year while on treatment, and safety concerns, such as the development of persistent high-titer neutralizing antibodies in patients receiving IFN-b therapy, among others. In the event of significant side effects that may be related to the medication, as well as in the case of women who get pregnant while undergoing treatment, many disease-modifying therapies necessitate the cessation of therapy. Only glatiramer acetate, which can be taken continuously throughout pregnancy, and in some cases previous use of ocrelizumab, alemtuzumab, and cladribine, which all have long-lasting pharmacodynamic effects that persist after the medication has been discontinued, are exempt from this restriction. Glatiramer acetate is a medication that can be taken continuously throughout pregnancy [59].

Association between vitamin D and therapeutic agents used in MS

Promising research by Hoepner et al. showed that 1,25-dihydroxyvitamin D3 enhanced the levels of Glucocorticoid Receptor (GR) protein in vitro, resulting in greater glucocorticoid-induced T cell death. The 1,25-dihydroxyvitamin D3/glucocorticoid combination treatment clinically improved the Experimental Autoimmune Encephalomyelitis (EAE) course more than the individual monotherapies, based on the T cell GR expression. In two MS cohorts, glucocorticoid-resistant relapses were linked to 1,25-dihydroxyvitamin D3 deficiency. The mTOR pathway, not the JNK pathway, was found to mediate synergistic 1,25-dihydroxyvitamin D3/glucocorticoid effects on induction of apoptosis. Inline, decreased 1,25-dihydroxyvitamin D3 levels in humans were associated with lower expression of mTORc1 inhibiting TSC1 in CD8+ T cells. GR activation by in vitro 1,25-dihydroxyvitamin D3 and 1,25-dihydroxyvitamin D3/glucocorticoid synergism and in vivo therapeutic effectiveness were eliminated in animals lacking T cell-specific mTORc1. Everolimus improved glucocorticoid effectiveness in EAE by inhibiting mTORc1. 1,25-dihydroxyvitamin D3 promotes glucocorticoid-mediated effects in T cells in vitro and in vivo via mTORc1 suppression. These findings may assist to boost anti-inflammatory glucocorticoid treatment [60].

A recent study conducted by Feng et al. showed increased expression of MxA and p-Y-STAT1 in monocytes, mononuclear, and T cells when vitamin D was supplemented, indicating that vitamin D boosted IFN responses in vitro, in both untreated and IFN-β-1b-treated MS. The study also showed that vitamin D administered along with IFN-β increased Th2 responses, and decreased Th17 and Th1 cytokines, thus reversing the Th1/th2 bias seen with IFN-β alone in MS. Both findings point to the potential benefits of the combined use of IFN-β and vitamin D in treating MS [61].

Among MS patients mainly being treated with beta-1b interferon, decreased vitamin D levels early on in the disease were associated with grave long-term progression and activity of MS while higher vitamin D levels correlated with slower progression rate and low MS activity [62]. However, a recent meta-analysis concluded that vitamin D supplementation (low or high dose) did not significantly affect disability and relapse rate in MS patients during treatment [63].

Outcomes of vitamin D supplementation on overall disease progression

A prospective cohort study carried out by Bhargava et al. revealed that on supplementation with vitamin D, there was a significant decrease in oxidative stress markers in healthy controls but not in patients with MS. Using metabolomics, they found metabolic alterations in xenobiotic metabolism and oxidative stress in patients with MS on vitamin D supplementation, demonstrating the use of metabolome studies in catching aberrant metabolic pathways and monitoring treatment response [64].

Research released by the University of Cambridge reported that vitamin D activates vitamin D nuclear receptor, which on pairing with retinoid X receptor (RXR-γ) leads to downstream signaling and increased differentiation of oligodendrocyte progenitor cells into mature oligodendrocytes that are crucial for myelin synthesis around neurons, and are often the damaged cells in MS. The study also showed vitamin D receptor expression in cells of oligodendrocyte lineage in MS, together with revealing the regenerative role of vitamin D in demyelinating diseases [65].

According to a study by Smolders et al, low vitamin D levels predicted higher exacerbation risk and MRI activity in those with early remitting-relapsing MS, with clinical trials on vitamin D supplementation reporting negative results on primary endpoints, the effect of supplementation of vitamin D on MS activity is less marked than that proclaimed by observational researches, and this may be due to confounding or reverse causality in those studies or may reflect trial design differences with respect to inclusion criteria, therapy utilized, primary and secondary outcomes powers, and vitamin D dose and duration of supplementation in clinical trials [66].

In a study undertaken by Ascherio et al., slower disease progression and lower MS activity were observed in those with high 25(OH)D levels while prognosis was very poor among those who had low levels of 25(OH)D when MS started, predicting 25(OH)D to be a powerful determinant of long-term disease progression and activity [62].

In a prospective cohort study on people with MS, self-reported supplementation with vitamin D was linked with a higher physical and mental quality of life cross-sectionally but only with an increased physical quality of life prospectively [67].

Two RCTs, the CHOLINE and SOLAR studies had successful effects on secondary endpoints concluding that supplementing vitamin D decreased the number of enlarging or new T2 lesions, new T1 lesions, and their annualized relapse rate, hypointense T1 lesion volume as well as disability progression. Current evidence supports that MS patients should prevent vitamin D deficiency, and aim for around 100 nmol/L or somewhat higher levels of vitamin D [66].

A literature review revealed that disease measures improved more in those with low baseline levels of vitamin D [68].

Raised blood levels of vitamin D have been correlated with brain volume preservation in patients with a condition called clinically isolated syndrome (CIS), a part of the disease course in MS, thereby mitigating post-CIS neurodegeneration and disability in the long term [69].

Daily recommended vitamin D intake for patients with MS

Recent studies have found that vitamin D3 supplemented at a dose of 10,400 IU was tolerable and safe in MS patients, and mediated pleiotropic immunomodulatory functions in vivo, such as reduction of memory effector CD4+ T cells, decreased production of IL-17 by CD4+ T cells and simultaneous central naïve and memory CD4+ T cells increase [70].

The recommended daily allowance (RDA) for vitamin D in infants is 10 mcg or 400 IU, from 1-70 years is 15 mcg or 600 IU, including pregnancy and lactation, and 70 years onwards is 20 mcg or 800 IU [70].

Vitamin D toxicity

At very high doses (like 130000 IU), vitamin D intoxication can occur presenting with muscle weakness, nausea, and vomiting, acute renal failure, and hypercalcemia, thus warranting a close eye for the potential dangers due to overdosing cholecalciferol [71]. Hypervitaminosis D often occurs with hypercalcemia.

It can also be seen in lymphomas and granulomatous disorders. A comprehensive clinical and pharmacological history is required for an early diagnosis of vitamin D toxicity. Vitamin D overdosing or too-frequent dose intervals for osteoporosis, osteomalacia, hypoparathyroidism, or renal osteodystrophy cause vitamin D toxicity in the majority of individuals. Vitamin D supplementation (including therapeutic dosages) has become commonplace in otherwise healthy people due to vitamin D's current prominence as a therapeutic agent for a variety of illnesses [72]. This might lead to an increased risk of intoxication; symptoms of which physicians must consider while prescribing vitamin D supplementation in chronic illnesses.

## Conclusions

In conclusion, understanding the structural and functional vitamin D roles is essential for the prevention and management of MS. The strong relationship between vitamin D and MS is a primary indication of the vital step of supplementation in preventing and managing MS. The contribution of causal variables, such as folate, vitamin B12, and homocysteine on the pathogenicity of MS is critical in devising appropriate approaches towards alleviating the health problem. Environmental factors related to vitamin D deficiency have proven to be central in triggering the onset and prognosis of the disease. Current treatment strategies for MS include steroids and plasmapheresis. The principal approach to MS, according to our review, must be to integrate dietary measures in the treatment protocols. Moreover, a multifaceted approach should therefore be in place to target the problem from various dimensions and enhance whole patient care. We recommend further studies to conduct RCTs to investigate the effectiveness of large dosage (1000 IUs/kg/day) vitamin D supplements on MS. But as of now, the role of moderate doses of vitamin D supplementation seems integral to the prevention and management of multiple sclerosis.
